# Exposures to Particulate Matter and Polycyclic Aromatic Hydrocarbons and Oxidative Stress in Schoolchildren

**DOI:** 10.1289/ehp.0901077

**Published:** 2009-12-14

**Authors:** Sanghyuk Bae, Xiao-Chuan Pan, Su-Young Kim, Kwangsik Park, Yoon-Hee Kim, Ho Kim, Yun-Chul Hong

**Affiliations:** 1 Department of Preventive Medicine, College of Medicine, Seoul National University, Seoul, Korea; 2 Department of Occupational and Environmental Health, Peking University School of Public Health, Beijing, China; 3 Department of Preventive Medicine, College of Medicine, Jeju National University, Jeju, Korea; 4 College of Pharmacy, Dongduk Women’s University, Seoul, Korea; 5 Department of Biostatistics and Epidemiology, Graduate School of Public Health, Seoul National University, Seoul, Korea; 6 Institute of Environmental Medicine, Seoul National University Medical Research Center, Seoul, Korea

**Keywords:** children, metal, oxidative stress, PAH, panel study, particulate matter

## Abstract

**Background:**

Air pollution is known to contribute to respiratory and cardiovascular mortality and morbidity. Oxidative stress has been suggested as one of the main mechanisms for these effects on health.

**Objective:**

The aim of this study was to analyze the effects of exposure to particulate matter (PM) with aerodynamic diameters ≤ 10 μm (PM_10_) and ≤ 2.5 μm (PM_2.5_) and polycyclic aromatic hydrocarbons (PAHs) on urinary malondialdehyde (MDA) levels in schoolchildren.

**Methods:**

The study population consisted of 120 schoolchildren. The survey and measurements were conducted in four cities—two in China (Ala Shan and Beijing) and two in Korea (Jeju and Seoul)—between 4 and 9 June 2007. We measured daily ambient levels of PM and their metal components at the selected schools during the study period. We also measured urinary 1-hydroxypyrene (1-OHP) and 2-naphthol, to assess PAH exposure, and MDA, to assess oxidative stress. Measurements were conducted once a day for 5 consecutive days. We constructed a linear mixed model after adjusting for individual variables to estimate the effects of PM and PAH on oxidative stress.

**Results:**

We found statistically significant increases in urinary MDA levels with ambient PM concentrations from the current day to the 2 previous days (*p* < 0.0001). Urinary 1-OHP level also showed a positive association with urinary MDA level, which was statistically significant with or without PM in the model (*p* < 0.05). Outdoor PM and urinary 1-OHP were synergistically associated with urinary MDA levels. Some metals bound to PM_10_ (aluminum, iron, strontium, magnesium, silicon, arsenic, barium, zinc, copper, and cadmium) and PM_2.5_ (magnesium, iron, strontium, arsenic, cadmium, zinc, aluminum, mercury, barium, and copper) also had significant associations with urinary MDA level.

**Conclusion:**

Exposure to PM air pollution and PAHs was associated with oxidative stress in schoolchildren.

Air pollution has been found to contribute to respiratory and cardiovascular morbidity and mortality, including asthma, myocardial infarction, stroke, and cancer (de Kok et al. 2006; Hong et al. 2002; Schwartz and Morris 1995). Although many epidemiologic studies have consistently shown the damaging health effects of air pollution, there are critical gaps in understanding the mechanism of such effects. Some animal studies have shown a pathogenetic linkage between exposure to air pollutants and health effects; however, evidence for human health is still limited (Møller et al. 2008).

Oxidative stress is known to be induced by air pollution in animal studies (Kodavanti et al. 2001), and epidemiologic studies conducted in urban areas have also demonstrated that oxidative stress was associated with environmental air pollutants in bus drivers (Rossner et al. 2008a). Oxidative stress is thought to be caused by redox-cycling organic chemicals and transition metals bound to the surface of particulate matter (PM) and produced by enzymatically catalyzed reactions in target cells. In general, PM with aerodynamic diameters ≤ 2.5 μm (PM_2.5_) has been observed to be more toxic than PM with aerodynamic diameters ≤ 10 μm (PM_10_) for the same unit increase in concentration, presumably with more chemicals bound to the particles (de Kok et al. 2006; Xia et al. 2006). Despite these findings, our understanding is still limited on whether PM air pollution causes oxidative stress in children.

Oxidative stress is defined as an impaired balance between free radical production and antioxidant capacity resulting in excess oxidative products (Hong et al. 2009). The generation of reactive oxygen species can cause oxidative damage to DNA, proteins, or lipids in the body. Malondialdehyde (MDA), which is an end product of the oxidation of polyunsaturated fatty acids and can determine the degree of lipid peroxidation, has been used as a marker for oxidative stress (Benzie 1996; Gutteridge and Halliwell 1990).

The aim of the present study was to analyze the effects of exposure to PM_10_ and PM_2.5_ and the metals bound to them, as well as urinary metabolites of polycyclic aromatic hydrocarbon (PAH) exposure, on urinary concentrations of MDA in schoolchildren.

## Materials and Methods

### Subjects

The study population consisted of 120 schoolchildren living in Ala Shan and Beijing, China, and in Seoul and Jeju, Korea. We selected one school in each city, and all study subjects of each city were recruited in the same school. Parents of each subject completed a questionnaire on demographics, past medical history, and environmental exposures. Exposure measurement and urine sample collections were conducted on 5 consecutive days between 4 and 8 June 2007 in Ala Shan and Beijing. However, because June 6th is a national holiday in Korea, the measurements were conducted in Seoul and Jeju between 4 and 9 June, excluding the holiday. The spot urine samples were collected at the end of class in the morning.

The study protocol was approved by the institutional review boards at Peking University and Seoul National University, and written informed consent was obtained from the parents of all study participants.

### Measurement of PM_2.5_, PM_10_, and metal components

We measured daily ambient concentrations of PM_2.5_ and PM_10_ in the schools for 24 hr/day during the study period. To obtain concentrations of PM_2.5_ and PM_10_, we gravimetrically analyzed dust on polytetrafluoroethylene filters. Metals from the collected PM_2.5_ and PM_10_ were determined using an inductively coupled plasma mass spectrometer (Perkin-Elmer, Waltham, MA, USA). Metal concentrations were calculated as the ratio of the metal amount in the PM_2.5_ or PM_10_ sample to the air volume collected during sampling. Measured metal components were strontium (Sr), arsenic (As), cadmium (Cd), zinc (Zn), mercury (Hg), barium (Ba), copper (Cu), lead (Pb), manganese (Mn), chromium (Cr), magnesium (Mg), iron (Fe), aluminum (Al), titanium (Ti), molybdenum (Mo), silicon (Si), boron (B), nickel (Ni), and zirconium (Zr), for both PM_2.5_ and PM_10_.

### PAH exposure markers 1-hydroxypyrene (1-OHP) and 2-naphthol (2-NAPH)

Morning urine samples were collected in conical tubes and sent to the laboratories in each city, and then aliquots were taken. This process took less than 1.5 hr. The samples were stored in the freezer at −20°C. To analyze urinary 1-OHP and 2-NAPH, urine samples were buffered with sodium acetate (pH 5.0) and hydrolyzed enzymatically using β-glucuronidase with sulfatase activity (Sigma, St. Louis, MO, USA) for 16 hr at 37°C in a shaking water bath (Jongeneelen et al. 1987; Kim et al. 1999). After hydrolysis, acetonitrile was added and samples were centrifuged at 10,000 × *g* for 10 min. A high-performance liquid chromatography (HPLC) system, consisting of a pump (Waters 600E; Millipore, Milford, MA, USA), a variable fluorescence detector (RF-10AxL; Shimadzu, Kyoto, Japan), an automatic injector (L-7200, Hitachi, Tokyo, Japan), and an integrator (Chromatopac C-R3A; Shimadzu) were used. A 150-mm-long reverse-phase column (TSK gel ODS-80Tm; Tosoh, Tokyo, Japan) was used to determine 1-OHP levels using a mobile phase of 58% (vol/vol) acetonitrile and excitation/emission wavelengths of 242/388 nm. For 2-NAPH analysis, a 250-mm-long reverse-phase column (J’sphere ODS-H80; YMC Inc., Wilmington, NC, USA) was used with a mobile phase of 38% (vol/vol) acetonitrile and 227/355 nm excitation/wavelengths.

### Oxidative stress biomarker MDA

Urinary MDA was determined by measuring MDA–thiobarbituric acid (TBA) adducts. Aliquots of the upper layer of the centrifuged urine sample were mixed with phosphoric acid and TBA reagent in an iced methanol glass tube and then boiled for 60 min. The mixture was then cooled in iced water for 5 min and centrifuged after the addition of methanol. The absorbance was measured at 532 nm after separation with an HPLC system equipped with an SP930D solvent delivery pump and UV730D absorbance detector (Younglin Co., Seoul, Korea) on a Nova-Pak C18 column (150 × 3.9 nm) with a mobile phase of 50 mM KH_2_PO_4_ (pH 6.8):methanol (58:42, vol/vol).

### Statistical analysis

City-specific analyses were performed using a linear mixed-effects model to estimate the effects of PM, metals, or PAH on oxidative stress. We conducted modified Levene test to determine homogeneity among the cities. Because there was heterogeneity of exposures among the cities, city-combined analyses were performed using a hierarchical linear mixed-effects model, where the city was treated as a random effect, to estimate the effects of exposures. We used the SAS mixed procedure (version 9.1.3; SAS Institute Inc., Cary, NC, USA) with compound-symmetry variance–covariance matrix. The effects were estimated after adjusting for individual variables such as age, sex, height, weight, environmental tobacco exposure, heating fuel, and city. We measured height and weight of each child. These variables and age were included as continuous variables in the models. We assessed environmental tobacco smoke by asking in the questionnaire whether there were smokers in the family. We also asked what kind of heating fuel they used. We included environmental tobacco smoke and heating fuel in the models as categorical variables. We treated each child as a random effect in the models, and the other variables were fixed effects. In analyses with multiple comparisons, the Bonferroni correction was applied. To compare the mean levels, we used the generalized linear model.

We assessed the interaction of exposures to PM and PAH using two methods. First, we included the interaction term in the mixed model. Second, we compared the mean level of MDA in different exposure groups. To do this, we categorized the exposures as greater or less than the 75th percentile. Exposures to PM exceeding 239.9 μg/m^3^ and urinary 1-OHP exceeding 1.42 μg/g creatinine were considered “high PM” and “high 1-OHP,” respectively. We compared the mean level of MDA according to the combination of these exposures. We also included the interaction term of these two dichotomized variables in the model.

## Results

Table 1 shows the basic characteristics of the study subjects. Mean (± SD) age ranged from 9.46 ± 0.51 years (Seoul) to 11.90 ± 0.31 years (Jeju). Mean height ranged from 133.73 ± 5.93 cm (Seoul) to 143.32 ± 6.49 cm (Beijing). Mean weight ranged from 31.22 ± 3.74 kg (Ala Shan) to 39.37 ± 8.76 kg (Jeju). Among the four cities, Ala Shan had the highest proportion of children with smokers at home (70%). Coal and oil were the main heating fuel in Ala Shan (70%) and Jeju (63%), respectively, and gas was the main heating fuel in Beijing (57%) and Seoul (73%).

Table 2 shows the mean levels of PM and least square means of biomarkers for PAH exposure and oxidative stress in each city. Mean concentrations of PM_2.5_ and PM_10_ were highest in Ala Shan and lowest in Jeju during the study period. Children from Ala Shan also showed the highest levels of biomarkers for PAH exposure and oxidative stress.

We evaluated the associations between urinary MDA levels and outdoor PM concentrations from the current day to the 2 previous days in each city. For 10-μg/m^3^ increments, levels of PM_10_ and PM_2.5_ on the previous day had positive associations in Ala Shan; we did not observe significant associations in the other cities (Table 3). However, when we combined the results for all the cities, we found significant associations between PM levels and urinary MDA concentrations from the current day to the 2 previous days. Considering the effect size of the associations and the data loss due to lag time, we used the exposure of 1 previous day in further analyses.

Table 4 shows the associations between the urinary PAH exposure and oxidative stress biomarkers. Because of the short half-lives of the biomarkers, we analyzed the associations between current-day levels of both biomarkers. We estimated effects for the increments of both 1-OHP and 2-NAPH by 1 μg/g creatinine. We found a significant association between 1-OHP and urinary MDA, whereas 2-NAPH was not significantly associated with the oxidative stress biomarker. Similar to the associations between PM and MDA, the city-specific analyses did not show significant associations, with the exception of 1-OHP in Ala Shan.

To identify interactive relationships between the outdoor PM concentrations and urinary 1-OHP levels with the oxidative stress biomarker, we conducted analyses with both exposure levels together in the models. We estimated effects for PM increments of 10 μg/m^3^ and 1-OHP increments of 1 μg/g creatinine. Table 5 shows the independent association of PM with the oxidative stress biomarker and urinary 1-OHP levels. Both 1-OHP and PM had significant associations with MDA when we included both exposures in the same model. However, the interaction term of both exposures was not significant in the relationship with urinary MDA levels.

For further analysis of the interaction of exposures to both PAHs and PM, we grouped subjects according to the levels of PM and urinary 1-OHP concentrations. The group with high PM_10_ exposure was identical to the group with high PM_2.5_ exposure, so we considered them as the same group highly exposed to PM. We compared the mean level of urinary MDA according to the extent of exposure controlling for age, sex, height, weight, heating fuel, and environmental tobacco exposure. The group with low exposure to both pollutants had the lowest mean level of MDA [0.91 μmol/g creatinine; 95% confidence interval (CI), 0.78–1.04]. The group with high exposure to PM (MDA level, 1.35 μmol/g creatinine; 95% CI, 1.09–1.61) and the group with high exposure to PAH (MDA level, 1.21 μmol/g creatinine; 95% CI, 0.99–1.42) had significantly higher mean levels of MDA than did the low-exposure group, but we found no significant difference between these groups. The group with high exposure to both pollutants had a significantly higher mean MDA level (2.11 μmol/g creatinine; 95% CI, 1.83–2.39) than any other group. The *p*-value for the interaction term of both exposures (*p* = 0.0197) is consistent with a synergistic interaction between PM and PAH on urinary MDA levels, so the estimated association for both exposures combined is greater than expected based on the associations estimated for high PM exposure alone and high PAH exposure alone.

**Figure 1:**
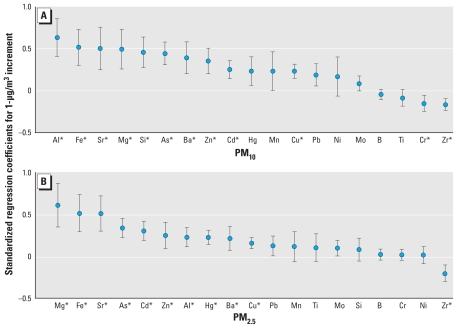
Associations of urinary MDA level with standardized concentrations of metal components of PM_10_ (*A*) and PM_2.5_ (*B*): standardized regression coefficients and 95% CIs, adjusted for exposure to environmental tobacco smoke and heating fuel in home, height, weight, city, sex, and age, with Bonferroni correction. Effects were estimated for the increment of 1 μg/m^3^ of each metal element, measured on the previous day. *p < 0.0025.

To evaluate the effects of metals bound to the PM on the oxidative stress biomarker, we conducted regression analyses with city-combined data and found extremely variable regression coefficients (data not shown). To compare the effects of different concentrations of metals, we standardized the levels of metal components by subtracting means of PM and dividing by standard deviation of PM of each city. We estimated effects for the increment of 1 μg/m^3^ of each metal element. We found significant positive associations of Al, Fe, Sr, Mg, Si, As, Ba, Zn, Cu, and Cd among PM_10_-bound metals at a significance level of 0.0025 after Bonferroni correction (Figure 1A). Mn, Hg, Pb, Ni, Mo, B, and Ti did not show significant associations. Among PM_2.5_-bound metals, we found significant positive associations of Mg, Fe, Sr, As, Cd, Zn, Al, Hg, Ba, and Cu with the oxidative stress biomarker (Figure 1B). Pb, Mo, Mn, Ti, Si, B, Cr, and Ni did not show significant associations. The level of Zr in both PM_10_ and PM_2.5_ showed negative association with urinary MDA levels.

## Discussion

The aim of this study was to investigate the association between exposure to PM or the metals bound to them and oxidative stress in schoolchildren. We also conducted analyses to investigate the association between PAH exposure and oxidative stress.

This study showed that ambient concentrations of PM_10_ and PM_2.5_ had significant associations with the urinary marker of oxidative stress in schoolchildren (Table 3). Exposure to PM has been shown to be associated with excessive respiratory and cardiovascular disease mortality and morbidity (Boezen et al. 1998; Burnett et al. 1995; Forsberg et al. 2005; Schwartz and Morris 1995; Schwartz et al. 1993). Studies have also shown significant associations between increased levels of PM and increased wheezing in infants and increased emergency department visits and admission for asthma in children (Andersen et al. 2008; Halonen et al. 2008; Jalaludin et al. 2008; Ko et al. 2007). One of the proposed mechanisms for these associations is that exposure to PM causes oxidative stress in humans (Becker et al. 2005; Nel 2005; Prahalad et al. 2001; Xia et al. 2006). A study conducted in Mexico showed that exposure to traffic-related PM had a positive relationship with increased oxidative stress and aggravated clinical outcomes in asthmatic children (Romieu et al. 2008).

In this study, we found significant associations between exposure to outdoor PM and urinary MDA levels in the city-combined analyses, whereas we found no significant association between these parameters in the city-specific analyses except in Ala Shan (Table 3). This reflects different levels of environmental exposure in the four cities and indicates a better exposure–response relationship in the city-combined than in the city-specific analyses because of the wider range of exposure and the larger sample size. Ala Shan not only had the highest level of PM but also had the highest proportion of children with exposure to environmental tobacco smoke and homes using coal as the heating fuel. These environmental exposures are thought to contribute to the level of PM and to the biomarkers for PAH exposure as well. However, other factors may have affected the antioxidant capacity of children, such as nutritional status (Elsayed 2001).

The different levels of exposure between cities may also have contributed to the similar results obtained on the association of urinary 1-OHP and MDA in the city-specific analyses, which did not show a significant association except in Ala Shan (Table 4). However, the city-combined analysis showed a significant association between urinary 1-OHP and MDA.

Urinary 1-OHP level has been shown to reflect exposure to PAHs in air pollution (Castaño-Vinyals et al. 2004; Cavanagh et al. 2007). Although we found that the level of urinary 1-OHP had a significant association with urinary MDA levels, we found no significant association between urinary 2-NAPH and the oxidative stress biomarker. The ambient level of outdoor 2-NAPH is known to vary depending on conditions, including sampling location and meteorologic conditions, so it may not accurately represent outdoor PAH levels (Preuss et al. 2003).

In the present study, children with exposure to both high levels of PAH and PM had significantly higher mean concentrations of urinary MDA than did children with high level of exposure to only one pollutant, or low levels of exposure. Furthermore, the difference in the mean level of urinary MDA between the group with both high exposures and those with one only high exposure was far greater than that between the groups with one high exposure or only low exposure. Both PAHs and PM are thought to be positively associated with oxidative stress, but little is known about the interactions between them (Becker et al. 2005; Hong et al. 2009; MohanKumar et al. 2008; Møller et al. 2008; Prahalad et al. 2001; Rossner et al. 2008a, 2008b; Xia et al. 2006). Our findings indicate that PAHs and PM have significant interactions with oxidative stress in schoolchildren.

PM comprises many components from various sources. Among these components, metals have been identified as a possible cause of respiratory diseases in children. In a previous study conducted in Korea, Pb and Mn were associated with decreased pulmonary function in schoolchildren (Hong et al. 2007). A previous *in vitro* study showed that transition metals such as Fe, Mn, Si, and Ti generated oxidative stress (Prahalad et al. 1999). In the present study, we found that concentrations of Fe, Si, and other metals bound to PM had significant associations with the oxidative stress marker MDA (Figure 1). Pb and Mn had positive associations but with marginal significance. This result shows that, in addition to the previously identified metals, various metals in PM were associated with oxidative stress and possibly contribute to clinical outcomes in schoolchildren. In contrast, Zr showed negative association with oxidative stress. Zr is known to inhibit the action of peroxidase (Ghosh et al. 1992), and this characteristic of Zr may have resulted in negative association observed in this study.

The strengths of the present study merit discussion. First, to the best of our knowledge, this is the first epidemiologic study to investigate the effects of outdoor exposure to PM and their metal components, controlling for PAH exposure, on oxidative stress in schoolchildren. Second, subjects participating in this study were from four different cities with a wide range of environmental exposure, which provided a good opportunity to evaluate exposure–response relationships. Third, by using biomarkers to evaluate exposures and outcome, more accurate evaluations were possible than in association studies using monitoring data only.

The present study also has some limitations. We measured ambient levels of PM in the schools of study subjects, and individual exposure could be different from the ambient PM levels. Individual exposures to other gaseous air pollutants such as nitrogen dioxide or ozone, and PM or PAHs in places other than the schools were not accounted for in this study. We assessed exposure to PAHs by using biomarkers, and these represent the whole exposure to PAHs from various sources and not limited to the PAHs exposures as component of PM. However, we used a questionnaire to obtain information about possible sources of air pollution at home for each child, such as environmental tobacco smoke and heating fuel, and adjusted these variables in the statistical analyses. In addition to exposure to air pollution, food intake is also known as an important route of exposure to PAHs. In this study, we did not assess exposure to PAHs from food intake. However, PAH exposure through food intake is related to dietary lifestyle, and the level of PAHs in food sources is thought to be consistent within the same area (Fiala et al. 2001). To control for different city-specific lifestyles and environments, we adjusted for the city factor in the city-combined analyses. Besides PAH exposures, these cities are different in many ways, such as culture, socioeconomic status, and populations. In addition, Jeju has a higher proportion of girls and higher mean age. We adjusted age, sex, and city in the analyses, but residual confounding may persist.

## Conclusion

In this panel study of schoolchildren in four cities, we observed that exposure to outdoor PM was associated with oxidative stress. This study also suggests that metals bound to PM are responsible, at least in part, for the oxidative stress. We found evidence of a synergistic effect of exposure to high levels of PM and PAH on oxidative stress. This provides new insight into the mechanism of the health effects caused by exposure to PM and PAH in children.

## Figures and Tables

**Table 1:** Basic characteristic of subjects by study areas.

Characteristic	Ala Shan	Beijing	Seoul	Jeju
Sex				
Male	17	15	16	4
Female	13	15	14	26
Age (years)	10.63 ± 0.56	10.13 ± 0.43	9.46 ± 0.51	11.90 ± 0.31
Height (cm)	140.43 ± 5.19	143.32 ± 6.49	133.73 ± 5.93	142.57 ± 6.63
Weight (kg)	31.22 ± 3.74	36.10 ± 5.44	34.16 ± 6.42	39.37 ± 8.76
Smoker in family	21 (70)	14 (47)	6 (20)	15 (50)
Heating fuel				
Coal	21 (70)	3 (10)	2 (7)	2 (7)
Electricity	0 (0)	2 (7)	2 (7)	5 (16)
Gas	1 (3)	17 (57)	22 (73)	3 (10)
Oil	1 (3)	0 (0)	4 (13)	19 (63)
Not available	7 (23)	8 (27)	0 (0)	1 (3)

Values are no., mean ± SD, or no. (%).

**Table 2:** Five-day mean ± SD levels of daily PM and least-square means (95% CI) of urinary biomarkers during the study period.

Measure	Ala Shan	Beijing	Seoul	Jeju	*p*-Value^a^
PM_2.5_ (μg/m^3^)	242.10 ± 134.97	146.70 ± 66.02	35.90 ± 23.54	15.30 ± 4.54	< 0.0001
PM_10_ (μg/m^3^)	409.50 ± 217.54	278.80 ± 80.90	52.90 ± 27.10	31.00 ± 9.44	< 0.0001
MDA (μmol/g creatinine)	1.74 (1.60–1.89)	1.00 (0.86–1.15)	1.17 (1.03–1.31)	0.90 (0.77–1.04)	< 0.0001
1-OHP (μg/g creatinine)	2.38 (2.22–2.55)	1.19 (1.03–1.36)	0.67 (0.51–0.83)	0.56 (0.40–0.72)	< 0.0001
2-NAPH (μg/g creatinine)	9.42 (8.48–10.36)	5.85 (4.93–6.77)	5.41 (4.51–6.32)	5.56 (4.66–6.47)	0.0504

a Modified Levene test for homogeneity of variance.

**Table 3:** Regression analyses of urinary MDA levels on PM_10_ and PM_2.5_ from current day to 2 previous days.

	MDA (mmol/g creatinine)		
Measure	**β**-Value	SE	*p*-Value
Ala Shan			
PM_10_			
Lag 0	− 0.0064	0.0054	0.2432
Lag 1	0.0120	0.0052	0.0242
Lag 2	0.0069	0.0050	0.1733
PM_2.5_			
Lag 0	− 0.0082	0.0114	0.4754
Lag 1	0.0281	0.0107	0.0100
Lag 2	0.0119	0.0097	0.2241

Beijing			
PM_10_			
Lag 0	0.0047	0.0093	0.6144
Lag 1	0.0131	0.0115	0.2582
Lag 2	− 0.0015	0.0098	0.8784
PM_2.5_			
Lag 0	0.0061	0.0129	0.6412
Lag 1	0.0165	0.0133	0.2157
Lag 2	0.0004	0.0113	0.9692

Seoul			
PM_10_			
Lag 0	0.0045	0.0250	0.8574
Lag 1	− 0.1234	0.1097	0.2628
Lag 2	− 0.0261	0.0252	0.3011
PM_2.5_			
Lag 0	0.0074	0.0273	0.7859
Lag 1	− 0.0358	0.1046	0.7326
Lag 2	− 0.0309	0.0267	0.2488

Jeju			
PM_10_			
Lag 0	− 0.1893	0.0554	0.0008
Lag 1	− 0.0801	0.0850	0.3483
Lag 2	7.5451	13.5564	0.5793
PM_2.5_			
Lag 0	− 0.2930	0.1198	0.0157
Lag 1	0.0716	0.1498	0.6335
Lag 2	− 0.1359	0.2442	0.5793

All cities			
PM_10_			
Lag 0	0.0156	0.0030	< 0.0001
Lag 1	0.0174	0.0046	< 0.0001
Lag 2	0.0137	0.0044	0.0020
PM_2.5_			
Lag 0	0.0275	0.0049	< 0.0001
Lag 1	0.0312	0.0070	< 0.0001
Lag 2	0.0250	0.0070	0.0004

Effects were estimated for 10-μg/m^3^ increments of PM. Each model was adjusted for city, age, sex, height, weight, environmental tobacco exposure, and heating fuel.

**Table 4:** Regression analyses of urinary MDA levels on PAH exposure biomarker concentrations in the urine of schoolchildren.

Biomarker (μg/g creatinine)	MDA (μmol/g creatinine)		
	**β**-Value	SE	*p*-Value
Ala Shan			
1-OHP	0.3724	0.0952	0.0002
2-NAPH	0.0921	0.1245	0.4611

Beijing			
1-OHP	− 0.0619	0.0858	0.4717
2-NAPH	− 0.0111	0.0987	0.9107

Seoul			
1-OHP	− 0.0079	0.3192	0.9803
2-NAPH	0.2042	0.2376	0.3999

Jeju			
1-OHP	0.0377	0.1171	0.7481
2-NAPH	− 0.0382	0.0636	0.5496

All cities			
1-OHP	0.0752	0.0309	0.0154
2-NAPH	0.0016	0.0065	0.8086

The biomarkers were measured on the same day. Each model was adjusted for city, age, sex, height, weight, environmental tobacco exposure, and heating fuel. Effects were estimated for the increments of both 1-OHP and 2-NAPH by 1 μg/g creatinine.

**Table 5:** Relationships of urinary 1-OHP levels and PM concentrations with urinary MDA levels and their interactions.

	MDA (μmol/g creatinine)		
Model	**β**-Value	SE	*p*-Value
Model 1			
1-OHP (μg/g creatinine)	0.0704	0.0303	0.0209
PM_10_ (μg/m^3^)	0.0017	0.0004	0.0001
1-OHP **×** PM_10_	0.0001	0.0002	0.6323
Model 2			
1-OHP (μg/g creatinine)	0.0729	0.0301	0.0161
PM_2.5_ (μg/m^3^)	0.0031	0.0007	< 0.0001
1-OHP **×** PM_2.5_	0.0003	0.0003	0.4332

Model 1 includes urinary 1-OHP and PM_10_. Model 2 includes urinary 1-OHP and PM_2.5_. Each model was adjusted for age, sex, city, height, weight, heating fuel, and environmental tobacco exposure. PM was measured on the previous day. Effects were estimated for the PM increments of 10 μg/m^3^ of PM and 1-OHP increments of 1 μg/g creatinine.
